# Federated fault diagnosis method for collaborative self-diagnosis and cross-robot peer diagnosis

**DOI:** 10.1371/journal.pone.0322484

**Published:** 2025-07-23

**Authors:** Yan Qin, Ouyang Wang

**Affiliations:** College of Engineering, ShaZhou Professional Institute of Technology, Zhangjiagang, Jiangsu, China; CINVESTAV IPN: Centro de Investigacion y de Estudios Avanzados del Instituto Politecnico Nacional, MEXICO

## Abstract

In multi-robot collaboration, individual failures can propagate to other robots due to the topological coupling between them. Existing fault diagnosis models are designed for single robots and fail to meet the practical requirements of multi-robot scenarios. To address this, this study develops a federated learning-based fault self-diagnosis model for individual robots and a multi-robot mutual diagnosis model that accounts for group behavior consistency. This approach effectively isolates faulty robots in multi-robot systems. Initially, each robot’s local data is encoded using the Gramian Angular Field (GAF) to generate two-dimensional time-frequency plots, creating local fault datasets. Next, a federated learning framework is established, where fault models for different robots are pre-trained using the local fault datasets. The local model parameters from multiple robots are then aggregated for shared learning, mitigating the potential knowledge shift during individual robot training. Finally, a multi-robot mutual diagnosis model is developed, incorporating group speed and direction consistency to ensure fault diagnosis based on behavioral coherence. Experimental results demonstrate that the proposed self-diagnosis model accurately identifies faults in individual robot components, while the mutual diagnosis model effectively recognizes system-wide faults.

## 1. Introduction

Due to the simplicity and distributed nature of multi-robot coordination, the system benefits from increased robustness and scalability to faults, offering significant advantages in tasks such as rescue operations, detection, and cargo handling [1]. However, in real-world multi-robot applications, robots may encounter failures for various reasons, including hardware malfunctions, software errors, sensor miscalibration, or environmental influences [2]. The diversity of these failure modes necessitates a fault diagnosis system capable of identifying and adapting to a wide range of potential failures. Therefore, ensuring effective information sharing and learning in the presence of faults and anomalies, while achieving accurate fault detection and diagnosis, represents a significant challenge for multi-robot systems [3].

Robot faults are typically identified as anomalies during the monitoring process. According to Gertler [4], monitoring systems can be categorized into fault detection, fault isolation (classification), and fault diagnosis. To effectively detect robot faults, it is necessary to analyze the fault signals, with common methods including parameter estimation and observer-based approaches. For instance, parameter estimation has been used to detect faults in robotic arms [5]. The core idea of observer-based methods is to estimate the system’s state output using known inputs and outputs. A common special case of state observers is the Kalman filter. Hasan et al. designed three model-based fault diagnosis algorithms, including a nonlinear adaptive observer (NLAO), an adaptive extended Kalman filter (AEKF), and an adaptive exogenous Kalman filter (AXKF), and compared their performance in a single-link robotic system [6].

Compared to analytical methods, data-driven approaches do not rely on mathematical models, as fault information is derived from the input data. The advantage of data-driven methods lies in their ability to transform high-dimensional data into low-dimensional spaces, effectively capturing essential information [7]. In particular, deep learning leverages its complex multi-layer network architecture to extract fault features hierarchically from robot fault data, offering new possibilities for feature extraction. This technique overcomes the challenges faced by traditional neural networks, such as incomplete fault samples, convergence issues, and non-real-time diagnosis [8]. Xia et al. combined multi-sensor data and utilized the structural advantages of convolutional neural networks (CNNs), taking into account both temporal and spatial information from raw data [9]. This automatic extraction of representative features eliminates the need for manual feature selection or expertise related to specific machinery and fault types. Pan et al. demonstrated the high accuracy and reduced training time of CNN-based methods for joint fault identification in robots, showing advantages over other techniques [10]. Additionally, Hamann et al. [11] combined deep networks with other methods to efficiently detect and diagnose faults, successfully implementing the approach on the pneumatic robot AirArm. Lu et al. designed a CNN model incorporating two different attention mechanisms, which extracted and fused robot fault features from multiple perspectives, resulting in superior performance [12]. Long et al. integrated sparse autoencoders and support vector machines (SVMs), achieving precise recognition of industrial robot fault patterns [13]. Miao et al. developed a multi-parameter fusion CNN model, introducing a weighting mechanism to enhance feature fusion, which was thoroughly validated using data from wheeled robots [14].

However, most existing fault diagnosis models are designed and constructed based on individual robots, neglecting the correlation between local and global knowledge within a robot cluster. This limitation reduces the effectiveness of fault diagnosis in multi-robot systems. In fault diagnosis for multi-robot systems, collaborative training across different robots’ data is essential. However, this raises significant data privacy concerns. Although these systems are often deployed within closed networks, data privacy remains a critical issue because sensitive information, such as operational states and locations, may expose the system’s security vulnerabilities. Moreover, in certain sensitive domains, such as military or public safety applications, data privacy is not only a matter of compliance but also of the system’s legitimacy and trustworthiness. Protecting the privacy of data shared between robots can enhance system reliability and foster greater collaboration among stakeholders. In contrast, federated learning enables collaborative model training across multiple entities without the need to exchange raw data directly [15,16]. This approach facilitates the creation of a unified, secure, and efficient multi-source data ecosystem, enabling large-scale data sharing across institutions. Li et al. proposed a diagnostic method using a federated learning framework, aggregating knowledge from multiple users to construct a global fault diagnosis model. The approach was validated by simulating scenarios where different users held limited fault data on rolling bearings under identical working conditions [17]. Zhang et al. introduced a method where the server dynamically aggregates model parameters to form a global fault diagnosis model, demonstrating high classification accuracy across two datasets [18]. To further enhance privacy, Li et al. developed a federated learning model with privacy-preserving trees, enabling collaborative fault diagnosis among industrial users while ensuring privacy protection [19]. They also proposed an autoencoder model to encourage participant contributions, resulting in improved model performance, which was validated on multiple bearing datasets. Considering the potential privacy risks from sharing weight parameters during model training, Wang et al. proposed a multi-level federated network based on interpretable metrics, constructing an adaptive sparse deep network and building a network association graph [20]. This method achieved effective fault diagnosis for ship bearings. Additionally, Lu et al. developed a federated learning framework for distributed wind turbine fault diagnosis, incorporating a gradient-based self-supervised scheme [21]. The proposed approach, validated on real wind turbine data, demonstrated high diagnostic performance while preserving data privacy.

In summary, this study establishes a joint diagnostic framework combining robot self-diagnosis and mutual diagnosis within a group. Within this framework, the mutual diagnosis model first analyzes differences in group behavior consistency to identify faulty individuals, enabling rapid fault detection. Subsequently, the self-diagnosis model is used to perform deep diagnostics on the faulty individual, ensuring accurate fault diagnosis. (1) In the self-diagnosis model, a federated learning-based distributed framework is employed to capture inter-robot relationships. This allows each robot to train a fault model using its local data and upload the model parameters to update the global model, thereby ensuring that the mutual influences between robots are fully considered and effectively facilitating fault diagnosis in individual robots within a cluster. (2) In the mutual diagnosis model, the group behavior is analyzed by assessing the speed and direction consistency among multiple robots to detect potential group-level fault states. Additionally, by calculating the deviation between each robot’s behavior and the average behavior of the entire group, specific faulty robots can be accurately identified, enabling effective fault detection and diagnosis across the multi-robot system. The contributions of this study are summarized as follows:

- A federated fault diagnosis framework combining self-diagnosis and mutual diagnosis was constructed, effectively identifying faults within the multi-robot system through information sharing and federated learning.
- The distributed self-diagnosis model captures the interdependencies among all robots, ensuring the accuracy of individual robot fault diagnosis.
- The mutual diagnosis model analyzes group behavior consistency, enabling rapid localization of faulty individuals within the multi-robot system, thereby enhancing the efficiency and precision of fault detection.

## 2. Research on behavior consistency-based mutual fault diagnosis method for multi-robot systems

### 2.1 Problem statement of behavior consistency in multi-robot systems

To address the challenge of utilizing individual interactions for fault diagnosis within multi-robot systems, a fault diagnosis method based on behavior consistency is proposed. This approach aims to identify faults by evaluating the behavior consistency among the members of the multi-robot system. Emphasizing the synchronization and coordination of behaviors among robots, the method employs comparative analysis of behavior patterns to assess whether the group may be experiencing a fault. Observations of significant inconsistencies or anomalies in behavior may indicate an abnormal state within the system. This strategy of diagnosing faults based on behavior consistency provides a solution for enhancing health monitoring, fault detection capabilities, and the overall reliability and operational efficiency of multi-robot systems.

During collaborative processes, each robot $r_{i}$ receives identical instructions and executes uniform actions. Theoretically, if all robots operate normally, their behaviors should demonstrate a high level of consistency. This consistency can be reflected in two aspects: speed consistency and direction consistency, meaning that each robot’s movement speed and direction should be synchronized with surrounding robots. If a robot experiences a fault, its motion system may be negatively affected, leading to discrepancies in its movement attributes. Therefore, by monitoring the consistency of speed and direction among the robots, it is possible to effectively diagnose whether the group is in a fault state.

Let *X* be a finite set of n robots in the Cartesian plane *Z*×*Z*, with each robot identified as $r_{i}\left(i\leq n\right)$ and $r_{i}\in X$. The system members $r_{i}$ possess the same capabilities for a given task; thus, if each robot $r_{i}\in X$ is treated equally without regard to identity, all robots are considered equal in the formation. Furthermore, the system members $r_{i}\in X$ are aware of one another $r_{j}\in X$, meaning the membership and number of system members are pre-known to all robots.

#### 2.1.1 Speed consistency.

The consistency of the robots’ movement rates is primarily observed in whether they maintain similar speeds under the same conditions, indicating coordination in their movement rates. In the case of speed inconsistencies caused by faults in the robot’s motion executors, attention is focused on changes in speed due to executor damage (either reduction or cessation of movement). Consequently, the distance traveled by the robots within a given command can be quantified to assess the state of the executors. Let the actual speed of each robot $R_{n}$ over a time period k be denoted as $D_{k}^{n}$. The speed consistency among the members can be calculated as:


$$SD_{k}=\sqrt{\frac{1}{n}\sum_{i=1}^{n}\left(D_{k}^{n}-\bar{D_{k}}\right)}$$ (1)

where $\bar{D_{k}^{n}}$ represents the average speed of all robots, calculated as shown in (2).


$$\bar{D_{k}}=\frac{1}{n}\sum_{i=1}^{n}D_{k}^{n}$$ (2)

In this equation, a smaller speed consistency $SD_{k}$ indicates that the movement speeds among robot members are more synchronized over the time period k, suggesting that the multi-robot system is functioning without faults. Conversely, a larger $SD_{k}$ implies potential abnormal behavior within the robot group.

#### 2.1.2 Direction consistency.

Direction consistency among the robots is primarily reflected in their ability to move toward a specified direction upon receiving identical instructions. In the multi-robot system, the direction of each robot $R_{n}$ can be characterized by a unit vector ${\vec{v}}_{k}^{n}$. To calculate the direction consistency between any two robots $R_{i}$ and $R_{j}$ with movement direction vectors ${\vec{v}}_{k}^{i}$ and ${\vec{v}}_{k}^{j}$, the following formula is used:


$$\mathrm{Cos}ineSimilarity\left({\vec{v}}_{i},{\vec{v}}_{j}\right)=\frac{{\vec{v}}_{k}^{i}\cdot {\vec{v}}_{k}^{j}}{\left\|{\vec{v}}_{k}^{i}\right\|\left\|{\vec{v}}_{k}^{j}\right\|}$$ (3)

where ${\vec{v}}_{i}\cdot {\vec{v}}_{j}$ denotes the dot product of the vectors, while $\left\|{\vec{v}}_{i}\right\|$ and $\left\|{\vec{v}}_{j}\right\|$ represent the magnitudes of the vectors.

Thus, the direction consistency of the multi-robot system can be characterized by computing the mean direction consistency of all robots:


$$DU_{k}=\frac{1}{n\left(n-1\right)}\sum_{i=1}^{n}\sum_{j=i+1}^{n}\mathrm{Cos}ineSimilarity\left({\vec{v}}_{k}^{i},{\vec{v}}_{k}^{j}\right)$$ (4)

where a larger direction consistency $DU_{k}$ indicates that the movement directions of the robots are more aligned, whereas a smaller $DU_{k}$ suggests greater directional deviations, indicating the potential presence of abnormal states within the robot group.

### 2.2 Behavior consistency-based mutual fault diagnosis model for multi-robot systems

In multi-robot systems, this study focuses on a specific scenario where all robots maintain a fixed formation, respond to identical control commands, and execute coordinated movements at consistent speeds and directions. In this context, a high degree of consistency among group members is essential for effective formation travel and synchronized operations. Therefore, when assessing the behavior consistency of multi-robot systems, two key indicators—speed consistency and direction consistency—are primarily considered. Speed consistency reflects the degree of coordination in movement speeds among the robots, while direction consistency assesses the alignment of movement directions among group members. When a multi-robot system demonstrates high consistency in these two metrics (Speed and direction consistency), it generally indicates strong collaborative capabilities, particularly in tasks that require coordinated movement, such as synchronized path following or collective navigation. However, in certain scenarios—such as when robots are required to avoid collisions, manipulate objects with different velocities, or explore distinct paths—robots may deliberately adopt different speeds and directions to optimize task completion. Therefore, high consistency in speed and direction is most applicable to scenarios where coordinated, synchronized movement is critical for achieving the task. Conversely, low consistency suggests the presence of abnormal behaviors within the group. To further diagnose which specific robot exhibits an anomaly, it is necessary to compare the observed behaviors with the expected behaviors of the group, thereby facilitating mutual diagnosis among the robots.

#### 2.2.1 Detection of abnormal states in multi-robot systems.

To effectively utilize speed and direction consistency for diagnosing faults in multi-robot systems, a consistency metric can be defined. This metric reflects the synchronization and coordination of behaviors among the robots. Let denote the number of robots in the system. At time **k**, the speed of each robot is denoted as $D_{k}^{n}$, and its direction is represented by a unit vector ${\vec{v}}_{k}^{n}$. The group behavior consistency can then be calculated as follows:

(1) Speed consistency:

$$SD_{k}=\sqrt{\frac{1}{n}\sum_{i=1}^{n}{\left(D_{k}^{n}-\bar{D_{k}}\right)}^{2}}$$ (5)

where $D_{k}^{n}$ is the speed of the n-th robot at time k and $\bar{D_{k}}$ is the average speed of the group at that time.

Additionally, to integrate direction consistency into the assessment of overall behavior consistency, the coefficient of variation of speed $VU_{k}$ can be utilized, as shown in Eq. (6):


$$VU_{k}=1-\frac{SD_{k}}{D_{k}}$$ (6)

When all robots have identical speeds, $VU_{k}$ approaches zero; as speed discrepancies increase, $VU_{k}$ becomes larger.

(2) Direction consistency:

$$DU_{k}=\frac{1}{n\left(n-1\right)}\sum_{i=1}^{n}\sum_{j=i+1}^{n}\mathrm{Cos}ineSimilarity\left({\vec{v}}_{i},{\vec{v}}_{j}\right)$$ (7)

When the directions of all robots are perfectly aligned, $DU_{k}$ is maximized; larger direction discrepancies lead to a decreased value of $DU_{k}$.

(3) Overall behavior consistency:

$$CU_{k}=\alpha \times VU_{k}+\left(1-\alpha \right)\times DU_{k}$$ (8)

where *α* is a weighting factor that adjusts according to the importance of speed consistency and direction consistency in specific applications.

Thus, the behavior consistency $CU_{k}$ comprehensively reflects the synchronization and coordination of the multi-robot system in terms of speed and direction. A larger $CU_{k}$ indicates greater stability within the group, while a smaller $CU_{k}$ suggests potential abnormal behaviors among the robots. Based on the comprehensive experimental analysis, the threshold for $CU_{k}$ is set to 0.85. Specifically, when $\text{0.85}\leq CU_{k}\leq \text{1}$, the multi-robot system is considered to be operating normally, while when $CU_{k}<0.85$, it indicates that the system is experiencing an anomaly.

#### 2.2.2 Individual fault diagnosis in multi-robot systems.

After identifying that faults exist within the multi-robot system, further steps can be taken to pinpoint which specific robot may be malfunctioning. This is achieved by comparing each robot’s behavior with the average behavior of the group. The average speed of the group is calculated as follows:


$$\bar{D_{k}}=\frac{1}{n}\sum_{i=1}^{n}D_{k}^{n}$$ (9)

where $D_{k}^{n}$ represents the speed of the *n*_th_ robot at time *k*. The average direction can be determined by computing the mean of the unit direction vectors.


$${\vec{v}}_{k}=\frac{1}{n}\sum_{i=1}^{n}{\vec{v}}_{k}^{n}$$ (10)

Consequently, the differences in behavior can be quantified, with speed deviation $\Delta D_{k}^{n}$ and direction deviation $\Delta v_{k}^{n}$ calculated as:


$$\Delta D_{k}^{n}=\left|D_{k}^{n}-\bar{D_{k}}\right|$$ (11)

$$\Delta v_{k}^{n}=1-\frac{{\vec{v}}_{i}\cdot {\vec{v}}_{j}}{\left\|{\vec{v}}_{i}\right\|\left\|{\vec{v}}_{j}\right\|}$$ (12)

To enhance the accuracy of diagnosing anomalies, thresholds for speed deviation $\theta_{v}$ and direction deviation $\theta_{d}$ can be established. If a robot’s $\Delta D_{k}^{n}>\theta_{v}$ or $\Delta v_{k}^{n}>\theta_{d}$ exceeds these thresholds, it is considered potentially faulty.

Therefore, utilizing this mutual diagnosis model enables effective detection of abnormal states within the group and facilitates the specific identification of malfunctioning robots, thereby achieving robust fault diagnosis for multi-robot systems.

## 3. Research on self-diagnosis method for individual robots based on federated learning

### 3.1 Federated averaging algorithm

**Fig 1:**
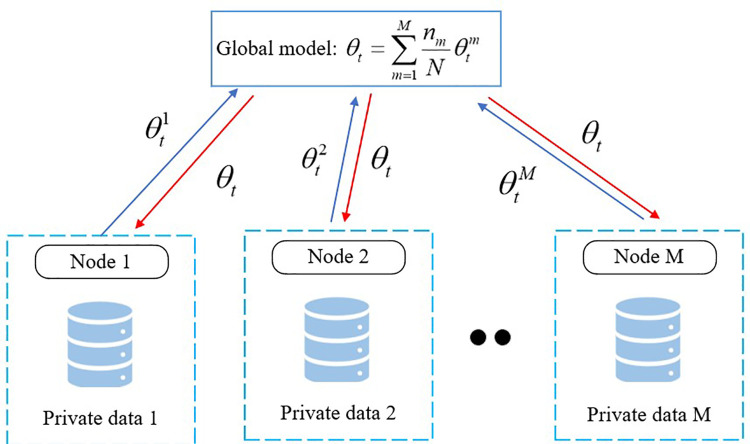
Federated averaging algorithm.

The federated averaging (FedAvg) algorithm is a distributed machine learning framework designed under the federated learning paradigm. It addresses the need for training models across multiple local datasets while preserving data privacy. The algorithm allows participants to independently update models on their local data using gradient descent, after which only the updated parameters are uploaded to a central server for aggregation. This process is iterated to achieve efficient global model training, as illustrated in **Fig 1**. Through this mechanism, data privacy is preserved, resource optimization is achieved, and the accuracy and generalization capability of the model are enhanced.

The process of the FedAvg algorithm can be summarized into three main stages. The first stage involves local model updates, where each client performs gradient descent on the received global model using its local dataset, thereby updating the local model parameters. In the second stage, the updated local model parameters and gradients are uploaded to a central server, where a weighted average is performed to generate the updated global model. The third stage consists of parameter distribution, in which the central server distributes the updated global model parameters back to all clients, allowing them to update their local models for the next training iteration. The detailed process is illustrated in **Algorithm 1**.

**Algorithm 1**. Federated averaging algorithm process.

Input: Initialized global model $\theta_{\text{0}}$

Output: Converged model after training $\theta_{t}$

1. The global training model $\theta_{\text{0}}$ is initialized by the central server and sent to all participating nodes.

2. Each node independently trains the model on its local dataset, adjusting the model parameters $\theta_{t}^{m}=\theta_{t-1}-\eta \nabla L_{m}\left(\theta_{t-1}\right)$ accordingly, where *η* represents the learning rate, and $L_{m}$ denotes the gradient value of the *m*-th node.

3. Upon completing local training, each node uploads its model parameters to the central server for synchronization.

4. The central server aggregates the parameters by calculating their average to update the global model $\theta_{t}=\sum_{m=1}^{M}\frac{n_{m}}{N}\theta_{t}^{m}$ , where $n_{m}$ represents the number of data samples at the *m*-th node, while *N* denotes the total number of samples across all clients.

5. The updated global model $\theta_{t}$ is then redistributed to the nodes for the next round of local training.

6. This process is repeated iteratively until the model converges.

### 3.2 Single robot fault self-diagnosis model

The one-dimensional fault signals are insufficient for extracting relative positional relationships when inputting into convolutional neural networks. Therefore, the GAF is employed to convert one-dimensional signals into two-dimensional images.

#### 3.2.1 Conversion of vibration signals to two-dimensional images based on Gramian Angular Field.

In the field of vibration signal analysis, transforming one-dimensional vibration signals into two-dimensional images represents an effective method for feature extraction and data analysis. The GAF is provided for encoding one-dimensional time series data into images. The primary concept involves scaling the one-dimensional sequence data and mapping the time series data to a polar coordinate system. By assessing the angles between different points, the temporal correlations among different time points are identified, and the relative angles between points in the sequence are computed to construct the image.

For a given time series *X* of the vibration signal, normalization is performed to ensure that all values are distributed within the interval [-1, 1], defined as follows:


$${\tilde{x}}_{t}=\frac{\left(x\left(t\right)-\max\left(X\right)\right)+\left(x\left(t\right)-\min\left(X\right)\right)}{\max\left(X\right)-\min\left(X\right)}$$ (13)

where $x_{i}$ represents the original vibration signal at each timestamp, ${\tilde{x}}_{t}$ is the normalized signal, and $\max\left(X\right)$ and $\min\left(X\right)$ are the maximum and minimum values in *X*, respectively. Consequently, values are encoded as the cosine of angles, and timestamps are represented as radii, allowing the normalized signal ${\tilde{x}}_{t}$ to be expressed in polar coordinates as follows:


$$\{\begin{array}{l} \theta_{i}=\arccos\left(x_{t}\right),-1\leq x_{t}\leq 1,x_{t}\in X\\ r_{i}=\frac{t_{i}}{N},t_{i}\in X \end{array})$$ (14)

where $t_{i}$ represents the timestamp and N is a constant factor for the normalized polar coordinate system’s generated space. Due to the bijective nature of this encoding process, when $-1\leq {\tilde{x}}_{t}\leq 11$, $\theta_{i}\in \left[0,\pi \right],\cos\left(\theta_{i}\right)$ maintains monotonicity within the specified range, ensuring the absolute mapping relationship between ${\tilde{x}}_{t}$ and $\theta_{i}$. Additionally, the radial coordinates and timestamps $t_{i}$ establish a linear relationship, thus preserving the absolute temporal relationships.

Based on the properties of trigonometric functions, the Gramian matrix can be classified into the Gramian Angular Sum Field (GASF) and the Gramian Angular Difference Field (GADF), defined as follows:


$$GASF=\left[\begin{array}{ccc} \cos\left(\theta_{1}+\theta_{1}\right) & \cdots & \cos\left(\theta_{1}+\theta_{n}\right)\\ \vdots & \ddots & \vdots \\ \cos\left(\theta_{n}+\theta_{1}\right) & \cdots & \cos\left(\theta_{n}+\theta_{n}\right) \end{array}\right]$$ (15)

$$GADF=\left[\begin{array}{ccc} \sin\left(\theta_{1}-\theta_{1}\right) & \cdots & \sin\left(\theta_{1}-\theta_{n}\right)\\ \vdots & \ddots & \vdots \\ \sin\left(\theta_{n}-\theta_{1}\right) & \cdots & \sin\left(\theta_{n}-\theta_{n}\right) \end{array}\right]$$ (16)

**Fig 2:**
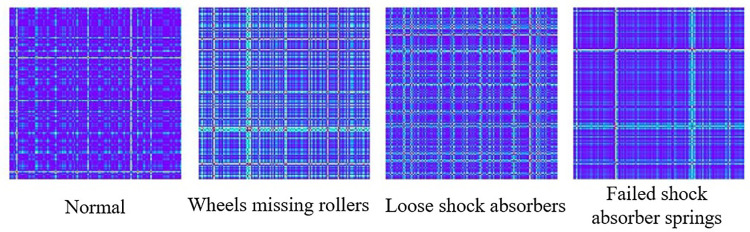
GASF images of different fault types.

In the context of fault diagnosis signal processing, GASF is advantageous as it retains the summative characteristics of signals, effectively highlighting periodicity or repetitiveness, making it suitable for a wide range of fault type diagnosis tasks. Therefore, this study employs the GASF method to transform vibration signals into two-dimensional images. The dataset’s vibration signals are segmented into multiple intervals, ensuring that each segment contains at least one complete cycle. These segments serve as inputs for the GASF method, generating a series of images representing different types of fault signal features, as illustrated in **Fig 2**, which are subsequently input into the CNN for fault diagnosis model training.

#### 3.2.2 Fault self-diagnosis model based on GAF-CNN.

For the fault diagnosis task of a single robot, this study employs a CNN to process the two-dimensional feature images encoded by the GAF, enabling precise diagnosis of motion actuator faults. By using images generated through the GASF method as input, the CNN model effectively identifies various types of faults. The core concept of the CNN is to utilize convolutional layers to extract features from the two-dimensional time-frequency images encoded by GAF. Dimensionality reduction and feature quantity reduction are achieved through pooling layers, followed by the integration of these features into the network’s output via fully connected layers. Let the weight at each position of the convolution kernel be represented as $w_{ij}$, and the corresponding convolutional region be denoted as $x_{ij}$. The convolution operation can be mathematically expressed as:


$$y=\sum_{i=1}^{h_{1}}\sum_{j=1}^{h_{2}}w_{ij}x_{ij}+b$$ (17)

where *y* represents the output value after convolution for each convolutional region, and *b* is the bias term.

**Fig 3:**
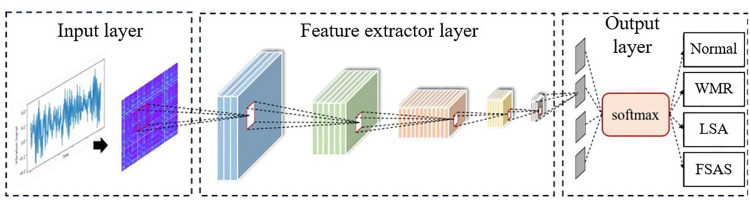
Structure of the GAF-CNN model.

The GAF-CNN model consists of three main components: the time-frequency image input layer, the fault feature extraction layer, and the diagnostic result output layer, as illustrated in **Fig 3**. The input layer utilizes GAF to convert vibration signals into two-dimensional image data. The feature extraction layer comprises convolutional layers, pooling layers, and fully connected layers. The convolutional layers employ multiple convolutional kernels to extract multi-dimensional feature maps from the input data, followed by nonlinear transformations using activation functions to enhance feature representation. The pooling layer reduces the size of the feature maps through down-sampling, thereby decreasing the computational burden while retaining critical feature information. This process employs small matrices as pooling windows, applying pooling operations to each segment of the feature map to gradually reduce its size while preserving the main features. The fully connected layer integrates all feature maps, mapping them to an output vector to accomplish the fault diagnosis task.

**Fig 4:**
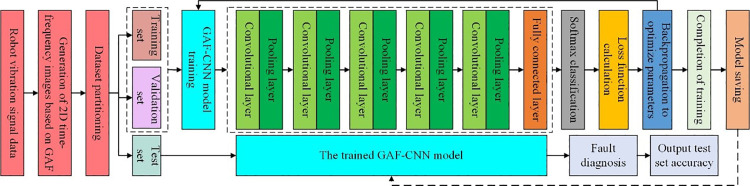
Training framework of the GAF-CNN model.

In the training process of the GAF-CNN model, the two-dimensional time-frequency images are first generated using GAF encoding, and the dataset is divided into training, validation, and testing subsets. Initially, the parameters of the CNN model are initialized. Subsequently, the GAF-CNN model is trained on the robot fault training set, while a pre-prepared test sample set is used to evaluate and test the model’s fault diagnosis performance. During the fault model training, the Softmax function is employed to classify the output layer into fault categories, ensuring that the predicted outputs align with the actual fault classes. Based on this error, the model parameters are optimized using the backpropagation algorithm to minimize the training objective. This iterative process continues until the model converges. The detailed training process of the GAF-CNN model is illustrated in **Fig 4**.

### 3.3 Federated learning-based self-diagnosis model for single robot faults

In the fault diagnosis tasks for multi-robot systems, the similarity in the operational principles and task execution among robot members results in consistency in data collection dimensions and features. Consequently, horizontal federated learning can be employed to collaborate across multiple robots by sharing the learning outcomes from sample data rather than the data itself. This approach facilitates the construction of a comprehensive global diagnostic model capable of addressing various operational conditions and disturbances. Such collaborative analysis not only enhances the accuracy and efficiency of fault diagnosis but also ensures the privacy and security of data across all robots, meeting the internal privacy protection requirements of the system.

**Fig 5:**
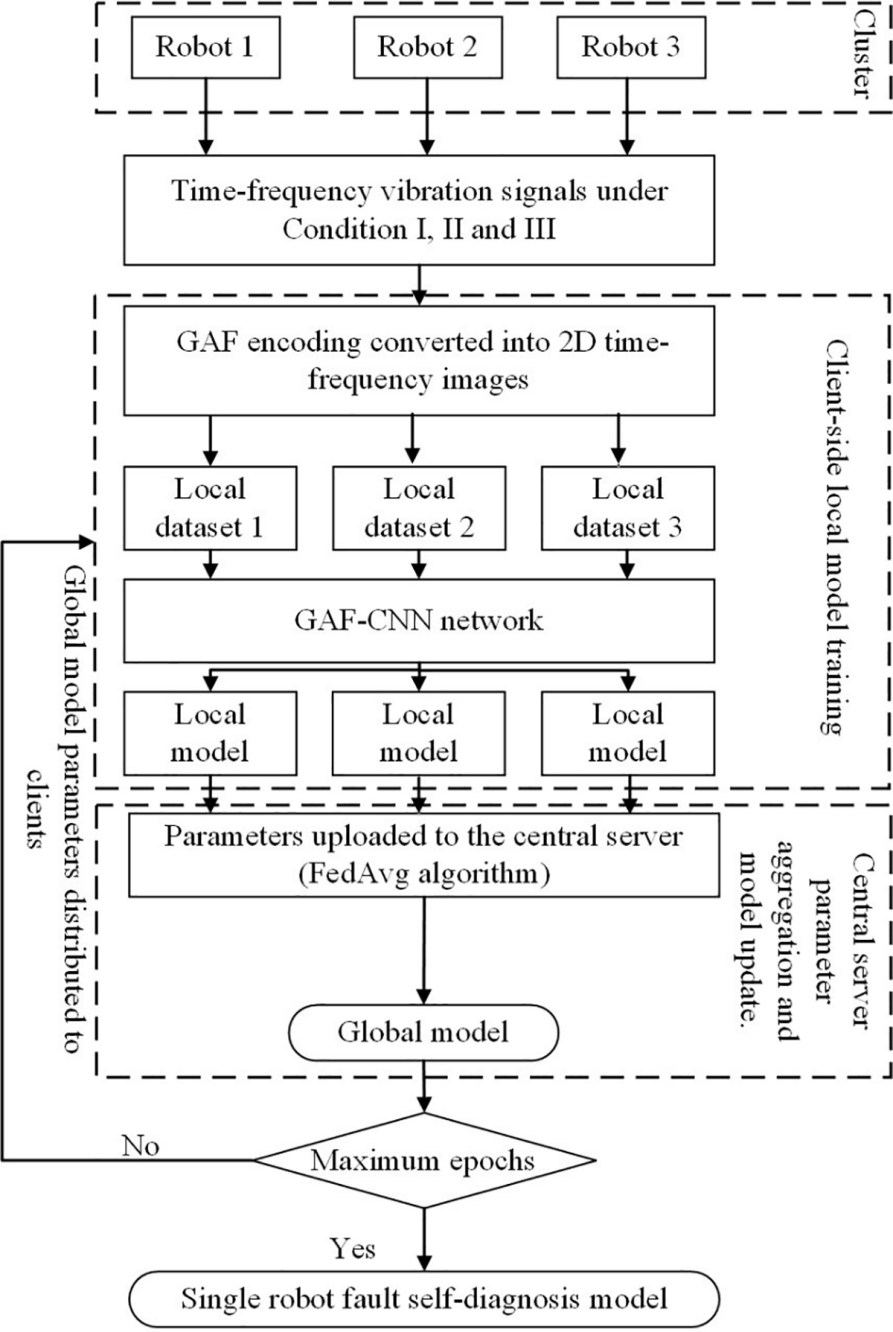
Workflow of federated learning-based fault diagnosis for multi-robot systems.

Horizontal federated learning requires overlapping features among the data across different robots, while local datasets vary among robots. In practical scenarios, vibration signal data from operational actuators of the multi-robot members are collected to construct local datasets under varying environments and conditions. Based on each robot’s local dataset, a local fault diagnosis model is trained using the GAF-CNN framework. The trained model parameters are then uploaded to a central computing node, where they are aggregated to update the global model. Finally, the updated model parameters are distributed to the corresponding robot nodes until the model converges, resulting in a self-diagnosis model applicable to individual robots and enabling rapid and accurate fault diagnosis across the multi-robot system, as illustrated in **Fig 5**.

In the multi-robot system, each robot trains a local model based on data generated during its actual operations. Thus, the local model reflects the fault diagnosis capabilities of the robot in its specific environment, showcasing its unique characteristics and advantages. Through the horizontal federated learning framework, the strengths of these local models can be integrated into a global model, enabling model capability aggregation. This process allows individual robots to share learning outcomes without exposing raw data, thereby safeguarding data privacy while enhancing the generalization performance of the global model by aggregating diagnostic capabilities from diverse environments. The specific steps of the federated learning-based fault diagnosis method are as follows:

In Step 1, data is collected from different operational conditions during the collaboration of multi-robot members, followed by preprocessing to improve data quality and usability. The GAF encoding technique is then employed to convert one-dimensional signals into two-dimensional images, thereby constructing each robot’s local dataset.

In Step 2, after setting the hyperparameters for the model, each robot node utilizes its local dataset to perform the E_th_ round of training on the initial GAF-CNN model. The objective is to extract critical fault features from the two-dimensional time-frequency images using convolutional neural networks, thus generating and optimizing their respective fault diagnosis models.

In Step 3, the robot nodes upload their local model parameters to the central computing node according to the specified compression rate, facilitating subsequent global model aggregation.

In Step 4, the central computing node employs the federated averaging algorithm to perform a weighted average of all uploaded local fault diagnosis model parameters, updating the global model. This aggregation step aims to integrate the training results from each robot to enhance the performance of the global model.

In Step 5, the aggregated global model parameters are redistributed to each robot node, allowing them to update their local fault diagnosis models based on the received global model parameters, thus maintaining model synchronization.

In Step 6, the new model parameters from Step 5 are distributed to promote optimization of the global model towards the target. Steps 2–6 are continuously repeated until the fault diagnosis model converges, yielding a global model for single robot self-diagnosis tasks.

## 4. Experimental validation of the fault self-diagnosis model

### 4.1 Dataset description

**Fig 6:**
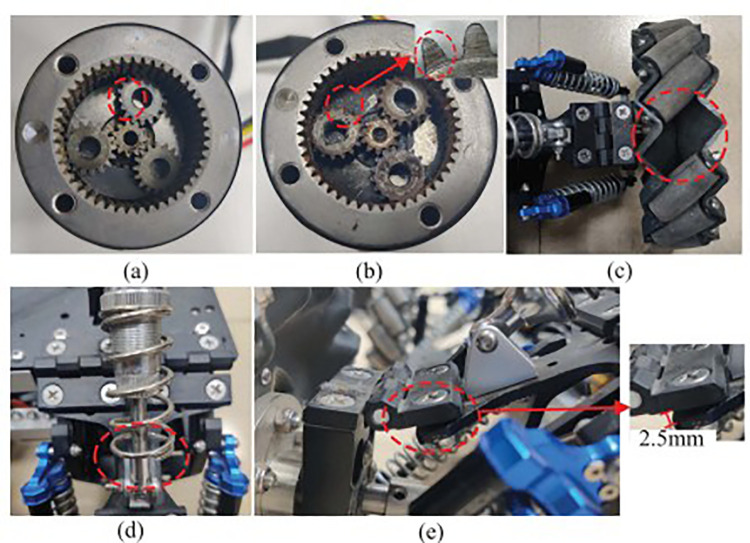
Robot fault modes.

The Shandong University Robot Dataset comprises high-quality data capturing five typical robot fault modes: gear tooth breakage in the motor gearbox, gearbox damage, wheel component loss, spring failure in the shock absorber, and loosening of the shock absorber [14]. These fault modes represent common issues encountered in critical mechanical components of robot systems. Table 1 summarizes the locations of the faults and their corresponding modes. Three operational speed settings for the robots were established: 0.5 m/s, 1.5 m/s, and 2.5 m/s. These settings simulate performance variations under different operational conditions. Additionally, the data sampling frequency was set at 100 Hz to capture sufficient detail. A schematic diagram of the fault configurations is presented in **Fig 6**, illustrating the specific component failures associated with each fault mode.

**Table 1:** Locations and modes of robot faults.

Label	Corresponding to the number in Fig 6	Fault location	Fault mode
S0	/	Normal	/
S1	(a)	Motor gearbox	Broken teeth
S2	(b)	Motor gearbox	Wear
S3	(c)	Wheel	Missing roller
S4	(d)	Shock absorber	Spring failure
S5	(e)	Shock absorber	Looseness

To simulate variability among individual robots, data collected at the three different speeds were treated as originating from different robots during their operational processes. Data processing was conducted for the five fault states: gear tooth breakage, gearbox damage, wheel component loss, spring failure, and shock absorber loosening. To preserve the temporal relationships within the data, a certain percentage of overlapping data was maintained during the time window segmentation, allowing for the capture of dynamic changes over time. Each sample segment was set to 2048 points, resulting in the extraction of 1000 samples from each group, culminating in a total of 6000 samples in the local dataset. To optimize the training efficiency of the local model, one-dimensional time series data from the local dataset were transformed into two-dimensional time-frequency images using GAF encoding, with corresponding labels assigned to these images. This transformation streamlined the data preprocessing process, accelerated model training speed, and retained crucial features of the data.

### 4.2 Experimental setup

#### 4.2.1 Hyperparameter configuration for the GAF-CNN model.

To effectively enable self-diagnosis of faults in individual robots, the selection of appropriate hyperparameters is crucial for the construction of the GAF-CNN model, as these parameters directly influence the model’s diagnostic accuracy. The hyperparameters primarily include filter size and quantity, dimensions of the fully connected layers, types of activation functions, classifier types, and learning rates based on the optimizer. A hyperparameter optimization approach was adopted in this study to construct the fault self-diagnosis model for individual robots, with the relevant structural parameters presented in **Table 2**.

**Table 2:** GAF-CNN model structural parameters.

Model parameter description	Value	Model parameter description	Value
Input image size	128×128×1	Pooling layer 4 filter size	2×2
Convolution layer 1 filter params	32×3×3	Convolution layer 5 flter params	32×3×3
Pooling layer 1 filter size	2×2	Pooling layer 5 filter size	2×2
Convolution layer 2 filter params	64×3×3	Fully connected layer 1 node count	64
Pooling layer 2 filter size	2×2	Dropout rate	0.2
Convolution layer 3 filter params	128×3×3	Number of output neurons	4
Pooling layer 3 filter size	2×2	Number of iterations	100
Convolution layer 4 filter params	64×3×3	Learning rate	0.001

#### 4.2.2 Federated learning model construction.

(1) Client setup

In the federated learning framework, each client undergoes initialization upon activation, which involves generating a unique client ID and loading the local training dataset. Once in the training environment, the client receives the initial model from the server and begins executing the training tasks. The client then batches data loading and model updates based on the configured hyperparameters. After completing the designated number of iterations, the client evaluates the discrepancies between local model parameters and server model parameters, uploading key parameter differences to the server for global model aggregation and updates. Client parameters are listed in **Table 2**.

(2) Server setup

Within the federated learning framework, the server is primarily responsible for model distribution, parameter aggregation, and performance evaluation on the test set. Upon server initialization, the test set and the models to be trained are loaded. During training, the central server prompts all robot nodes to participate in training the fault diagnosis model and disseminates the initial GAF-CNN network model parameters. After completing the training, robot nodes upload their model parameters to the server, which aggregates these parameters using the FedAvg method to update the global model. The updated model is validated on the test set to assess the performance of the aggregated global model for that round. This process is repeated until the predetermined number of aggregation rounds is reached, with relevant parameter settings detailed in **Table 3**.

**Table 3:** Model training parameters under the federated learning framework.

Parameter description	Value
Federated iterations	30
Layer compression rate	1.00337

### 4.3 Experimental results and analysis

The primary objective of the designed experiment is to validate the effectiveness of the fault self-diagnosis model in detecting faults in robot motion execution mechanisms. The experiment entails training three fault diagnosis models and one federated model. Each of the three fault diagnosis models is tested independently on its corresponding robot fault dataset to confirm that the foundational GAF-CNN model can accurately diagnose actuator faults. Additionally, the federated learning framework is utilized to conduct joint learning using the three local datasets, training a federated GAF-CNN fault diagnosis model.

To comprehensively evaluate the model’s performance, two key metrics—accuracy and recall—are employed. Accuracy measures the model’s ability to correctly diagnose faults, while recall assesses the proportion of actual faults identified by the model. The test set is constructed by proportionally sampling from the datasets under three operating conditions to further evaluate the model’s fault diagnosis performance.

#### 4.3.1 Fault diagnosis model experiment without federated learning framework.

To assess the effectiveness of the federated learning framework, a control group was established. In this group, each robot’s independent local data was utilized for model training, followed by the evaluation of each model’s accuracy using an existing test set. The experimental results are summarized in **Table 4**.

**Table 4:** Experimental results of robot fault diagnosis model (Without federated learning).

Fault type	Local model 1		Local model 2		Local model 3	
	Accuracy	Recall	Accuracy	Recall	Accuracy	Recall
Normal	98.15	98.4	97.87	98.5	98.62	100
Gearbox tooth breakage		98.2		98.0		98.4
Gearbox damage		98.0		97.6		98.3
Wheel missing roller		97.5		97.7		98.8
Damper spring failure		97.8		98.3		98.2
Damper looseness		99.0		97.1		98.0

#### 4.3.2 Fault diagnosis model experiment with federated learning framework.

This experiment aimed to validate the application effectiveness of federated learning in fault diagnosis models. By aggregating the local model parameters from each robot within the group, the global model was jointly optimized. After multiple iterations, the results demonstrated that federated learning enabled each participating robot to more effectively identify and diagnose various fault patterns. The specific results of the experiment are presented in **Table 5**.

**Table 5:** Experimental results of robot fault diagnosis model (With federated learning).

Fault type	Accuracy (%)	Recall (%)
Normal	99.33	100
Gearbox tooth breakage		100
Gearbox damage		100
Wheel missing roller		99.2
Damper spring failure		98.8
Damper looseness		98.0

A comparison of the local model and the model trained using federated learning revealed a significant enhancement in the diagnostic accuracy of the federated model across six fault types, reaching 99.33%. This represents an improvement of approximately 0.7% to 1.5% over the local models, highlighting the potential and effectiveness of federated learning in enhancing self-diagnostic capabilities among robots. The results indicate that the federated learning-based diagnostic model can leverage operational and fault data from various robots, enabling the model to gain a more comprehensive understanding of diverse fault conditions and improving its generalization ability. Particularly in multi-robot systems, where different operational environments may lead to varied fault characteristics, federated learning integrates the unique aspects of each dataset while preserving the privacy of individual datasets, thus improving the accuracy and efficiency of fault diagnosis.

#### 4.3.3 Comparison with existing algorithms.

To better assess the performance improvements of the constructed self-diagnostic model, the experimental control group included comparisons with representative existing algorithms, as summarized in **Table 6**.

**Table 6:** Comparative results with existing representative algorithms.

Fault type	LSTM-CNN		CWT-CNN		GAF-CNN		Proposed Method	
	Accuracy	Recall	Accuracy	Recall	Accuracy	Recall	Accuracy	Recall
S0	96.62	97.2	97.92	97.2	98.18	98.2	99.33	100
S1		96.7		98.4		98.1		100
S2		95.5		98.3		98.0		100
S3		97.0		98.7		99.2		99.2
S4		96.4		98.1		97.6		98.8
S5		96.9		96.8		98.0		98.0

The experimental results indicate that all CNN-based neural network models achieved accuracy and recall rates exceeding 95%, demonstrating strong baseline performance and confirming the effectiveness of CNNs in processing time-series data. By incorporating long short-term memory networks (LSTM), continuous wavelet transform (CWT), and GAF encodings into the CNN models, diagnostic performance improved, with the GAF-CNN model exhibiting the most significant enhancement. Notably, the GAF-CNN model employing federated learning outperformed the other models, achieving an accuracy of 99.33%. This indicates that federated learning effectively enhances the model’s generalization performance, making it particularly suitable for fault diagnosis tasks in multi-robot systems.

**Fig 7:**
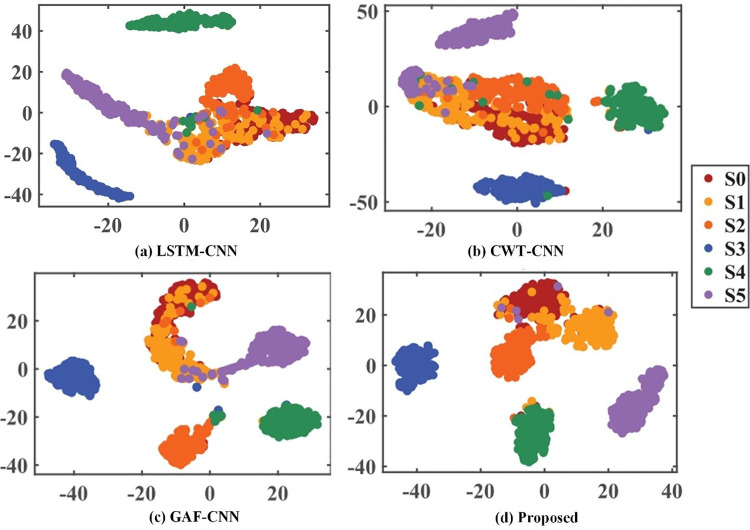
Comparison of feature extraction results of four methods.

To clearly illustrate the feature extraction effectiveness of each method, t-distributed stochastic neighbor embedding (t-SNE) was employed to visualize the output features of the LSTM-CNN, CWT-CNN, GAF-CNN, and the proposed method. The visualization results are shown in **Fig 7**.

As seen in **Fig 7(a)**, the LSTM-CNN exhibited a high degree of feature mixing, with poor clustering among categories, suggesting that irrelevant and redundant information led to relatively low feature discernibility. **Fig 7(b)** and **7(c)** indicate that both methods still exhibited significant feature overlap. In contrast, **Fig 7(d)** illustrates that the proposed method achieved effective clustering for each fault pattern, with a notable reduction in overlap issues, indicating its ability to extract more distinguishable features.

## 5. Cooperative fault diagnosis model for multi-robot systems

### 5.1 Experimental platform setup

**Fig 8:**
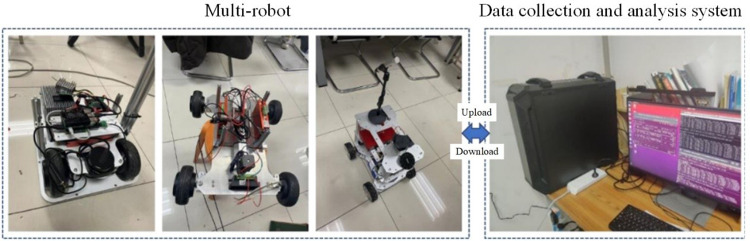
Experimental platform for the cooperative fault diagnosis model in multi-robot systems.

In the experiment, a multi-robot system was constructed using three robots with similar functionalities to establish an appropriate testing environment. These robots were equipped with identical hardware components, including speed sensors and directional control units, to ensure accurate measurement and control of their movement. To effectively capture the vibration signals from the actuators of the multi-robot system, a data acquisition system is designed for sensor data collection and analysis, as shown in **Fig 8**. The system uses an accelerometer (PCB Piezotronics 356A16 three-axis accelerometer) to capture the vibration signals. Signal conditioning and data acquisition are performed through the NI cDAQ-9174 data acquisition chassis and the NI 9234 acquisition module. The system is configured with a 16-bit resolution and a sampling frequency set to 100 Hz, which meets the vibration signal acquisition requirements within the 0.5–5000 Hz frequency range.

During the experiment, speed and gyroscope sensors provided real-time data on each robot’s velocity and directional parameters. Using the cooperative fault diagnosis model, the consistency among the robots was calculated, enabling the detection of any faults within the system. Hardware environment: Three inspection robots, speed sensors, gyroscope sensors, data acquisition devices, workstation with CPU (i9-9900k @ 3.6GHz), two GPUs (RTX-4090), and a 2TB solid-state drive. Software environment: Ubuntu 20.04 operating system, Python programming language, PyCharm development environment.

### 5.2 Experimental setup

The experiment was conducted using three robots equipped with basic mobility capabilities and data transmission functions. Each robot was outfitted with speed and gyroscope sensors, along with the necessary communication devices. The experiment took place in a flat, open indoor environment to minimize external interference. The task involved the robots following a predetermined path, such as moving in a straight line, with each robot programmed to move at specific speeds and directions. The robots communicated their position and speed information through a wireless system.

**Fig 9:**
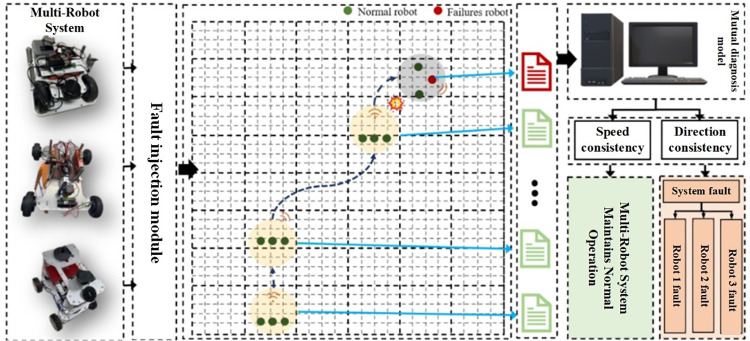
Experimental process of the cooperative fault diagnosis model for multi-robot systems.

In this experiment, the performance of the cooperative fault diagnosis model was tested by having the robots maintain a specific formation (e.g., linear or triangular) while transmitting their speed and direction data to a central data recording system, as depicted in **Fig 9**. To simulate real-world fault scenarios, disturbances were injected into the system by introducing predefined types of faults to specific robots at designated times. This method effectively tested the model’s ability to maintain formation stability and respond to faults.

The experimental setup demonstrated not only the practical applicability of the cooperative fault diagnosis model but also validated its effectiveness in diagnosing faults within a multi-robot system.

To further evaluate the effectiveness of this method in diagnosing faults in multi-robot systems and to assess the impact of operational consistency on fault diagnosis, ten comparison experiments were conducted. These experiments examined the effects of different fault types and the number of faults on the model’s performance. Detailed experimental settings are provided in **Table 7**.

**Table 7:** Experimental settings for the cooperative fault diagnosis model in multi-robot systems.

Number	Test subject	Fault mode	Number of faulty robots	Number of trials	Completion condition
1	Robots R1/R2/R3	No failure	0	50	60s
2	Robots R1/R2/R3	Speed deviation	1	50	60s
3	Robots R1/R2/R3	Speed deviation	2	50	60s
4	Robots R1/R2/R3	Speed deviation	3	50	60s
5	Robots R1/R2/R3	Direction deviation	1	50	60s
6	Robots R1/R2/R3	Direction deviation	2	50	60s
7	Robots R1/R2/R3	Direction deviation	3	50	60s
8	Robots R1/R2/R3	Speed and direction deviation	1	50	60s
9	Robots R1/R2/R3	Speed and direction deviation	2	50	60s
10	Robots R1/R2/R3	Speed and direction deviation	3	50	60s

To evaluate the diagnostic performance of the proposed multi-robot mutual diagnosis model, four metrics are introduced: Anomaly Detection Accuracy (ADA), True Positive Rate (TPR), False Positive Rate (FPR), and Fault Diagnosis Accuracy (FDA). The formulas for each of these metrics are as follows:


$$\text{ADA}=\frac{TP+TN}{TP+TN+FP+FN}$$ (18)

$$\text{TRP}=\frac{TP}{TP+FN}$$ (19)

$$\text{FPR}=\frac{FP}{FP+TN}$$ (20)

$$\text{FDA}=\frac{\text{Number of correct positioning}}{\text{Actual number of failures}}$$ (21)

where *FP* represents the number of times the model incorrectly identifies a fault when the system is operating normally; *TP* refers to the number of times the model correctly identifies a fault when the system is faulty; *FN* indicates the number of times the model incorrectly identifies the system as normal when it is actually faulty; and *TN* represents the number of times the model correctly identifies the system as normal when it is operating normally.

### 5.3 Experimental results and analysis

This experiment primarily focuses on validating the effectiveness and accuracy of the multi-robot mutual diagnosis model under normal system states, single fault modes, and combined fault modes. The experimental results demonstrate that the anomaly detection accuracy is consistently high across all scenarios, indicating that the model can reliably detect whether the system is experiencing anomalies, regardless of the fault mode or the number of faults. When an anomaly is present, the mutual diagnosis model performs well in diagnosing specific faulty robots, particularly in handling compound faults. The experimental results are shown in **Table 8**.

**Table 8:** Experimental results of the multi-robot mutual diagnostic model.

Number	Fault mode	ADA	TRP	FRP	FDA
1	No failure	100%	100%	0%	100%
2	Speed deviation	100%	100%	0%	96.0%
3	Speed deviation	100%	100%	0%	78.0%
4	Speed deviation	100%	100%	0%	70.0%
5	Direction deviation	100%	100%	0%	98.0%
6	Direction deviation	100%	100%	0%	76.0%
7	Direction deviation	100%	100%	0%	72.0%
8	Speed and direction deviation	100%	100%	0%	100%
9	Speed and direction deviation	100%	100%	0%	84.0%
10	Speed and direction deviation	100%	100%	0%	74.0%

Specifically, the anomaly detection accuracy remains at 100% across all test scenarios, demonstrating the high reliability and stability of this detection method. This consistency indicates that the model is highly sensitive and accurate in the initial identification of machine states, validating the effectiveness of the anomaly detection approach. When the multi-robot system experiences an anomaly, under a single fault mode (speed or direction deviation), the fault diagnosis accuracy remains above 70% even as the number of faults increases, suggesting the model’s strong ability to identify fault types. Notably, when only one robot is faulty, the diagnosis accuracy reaches 96% to 98%, reflecting the model’s effectiveness in identifying and addressing single fault types. In the combined fault mode, compared to the single fault mode, the diagnosis accuracy for faulty robots improves by 2% to 8%, indicating that the mutual diagnosis model can extract information from more dimensions of indicators. As a result, the combined fault types (speed and direction faults) consistently demonstrate higher diagnostic accuracy across all scenarios, suggesting that the diagnostic method exhibits robustness in handling more complex fault modes.

Overall, these experimental results validate the effectiveness of the multi-robot system’s mutual diagnosis model. The model demonstrates strong identification and handling capabilities for different types and complexities of faults, particularly exhibiting perfect accuracy during the anomaly detection stage. This lays a solid foundation for the future development of multi-robot fault diagnosis research.

## 6. Conclusion

To address the challenge of fault correlation in multi-robot systems, where it is difficult to directly diagnose group-level faults, a hybrid approach combining mutual diagnosis within the group and self-diagnosis at the individual level was proposed. This method identifies specific faulty robots by analyzing discrepancies in group behavior consistency and subsequently applies a self-diagnostic model to diagnose the fault, thereby achieving efficient fault diagnosis in multi-robot systems. In the self-diagnosis model, a federated learning-based distributed framework was adopted to capture both local and global fault correlations within the multi-robot system. This allowed individual robots to train fault models on local data and upload updated model parameters to a global model, ensuring that inter-robot interactions were fully considered. This method was particularly effective in diagnosing component faults in individual robots within the group. In the mutual diagnosis model, robots’ speed and direction changes were compared to assess their behavior consistency with the group, effectively detecting faults in the system. Further, by analyzing deviations between individual and group behaviors, the model accurately identified faulty robots, enabling rapid fault recognition in the multi-robot system.

Future research should focus on group-level fault diagnosis, exploring how to identify and predict potential faults from group dynamics.
